# ERRATUM

**DOI:** 10.1590/0074-02760230001er01

**Published:** 2023-02-20

In the article “**DNA sequences of *Toxoplasma gondii* for development of diagnostic probes**”, DOI number: 10.1590/s0074-02761991000400023, published in *Mem Inst Oswaldo Cruz*, Rio de Janeiro, Vol. 86(4), 1991, on page 483:

Where it reads:

Viviana Pzsenny

It should read:

Viviana Pszenny

